# Temporal Trends of In-Hospital Mortality in Patients Treated with Intra-Aortic Balloon Pumping: A Nationwide Population Study in Taiwan, 1998-2008

**DOI:** 10.1371/journal.pone.0131575

**Published:** 2015-06-26

**Authors:** Chung-Han Ho, Zhih-Cherng Chen, Chin-Chen Chu, Jhi-Joung Wang, Chun-Yen Chiang

**Affiliations:** 1 Department of Medical Research, Chi-Mei Medical Center, Tainan City, Taiwan; 2 Department of Hospital and Health Care Administration, Chia Nan University of Pharmacy and Science, Tainan City, Taiwan; 3 Division of Cardiology, Department of Internal Medicine, Chi-Mei Medical Center, Tainan City, Taiwan; 4 Department of Anesthesiology, Chi-Mei Medical Center, Tainan City, Taiwan; 5 Department of Pharmacy, Chia Nan University of Pharmacy and Science, Tainan City, Taiwan; San Raffaele Scientific Institute, ITALY

## Abstract

Intra-aortic balloon pumping (IABP) is widely used for hemodynamic support in critical patients with cardiogenic shock (CS). We examined whether the in-hospital mortality of patients in Taiwan treated with IABP has recently declined. We used Taiwan’s National Health Insurance Research Database to retrospectively review the in-hospital all-cause mortality of 9952 (7146 men [71.8%]) 18-year-old and older patients treated with IABP between 1998 and 2008. The mortality rate was 13.84% (n = 1377). The urbanization levels of the hospitals, and the number of days in the intensive care unit, of hospitalization, and of IABP treatment, and prior percutaneous coronary intervention (PCI) were associated with mortality. Seven thousand six hundred thirty-five patients (76.72%) underwent coronary artery bypass grafting (CABG) surgery, and 576 (5.79%) underwent high-risk PCI with IABP treatment. The number of patients treated with IABP significantly increased during this decade (p_trend_ < 0.0001), the in-hospital all-cause mortality for patients treated with IABP significantly decreased (p_trend_ = 0.0243), but the in-hospital all-cause mortality of patients who underwent CABG and PCI plus IABP did not decrease. In conclusion, the in-hospital mortality rate of IABP treatment decreased annually in Taiwan during the study period. However, high-risk patients who underwent coronary revascularization with IABP had a higher and unstable in-hospital mortality rate.

## Introduction

Intra-aortic balloon pumping (IABP) is the most widely used circulatory assistance method for patients with cardiogenic shock (CS). It can increase myocardial and peripheral perfusion and reduce afterload and then improve cardiac output. It is also used in patients being weaned from a cardiopulmonary bypass (CPB). A recent meta-analysis [1] reported that preoperative and 30-day mortality was significantly lower in patients who underwent coronary artery bypass graft (CABG) plus perioperative IABP. IABP is sometimes used in ventricular arrhythmia and high-risk percutaneous coronary intervention (PCI) [2]. Using IABP in thrombolytic therapy increases the survival rate of patients with acute myocardial infarction (AMI) and CS [3,4]. Thus, IABP is an intervention that might reduce perioperative mortality [5,6]. However, the IABP-Shock II trial [7,8], in which outcomes were followed-up for ~1 year, showed no reduction in 30-day mortality in patients with AMI and CS who also underwent early PCI. In the treatment guidelines for an ST-segment myocardial infarction (STEMI) with CS, IABP was a IIB indication in Europe [9] and a IIA indication in the USA [10].

Benchmark Registry data from 1996 to 2000 [11] showed that the in-hospital mortality in patients treated with IABP was 21.2%. A recent study [12] from a USA database said that, for patients treated with IABP and a PCI, there was a temporal reduction in all-cause mortality from 1.1% in 1998 to 0.86% in 2008. However, it is unknown whether there has been a recent decline in the in-hospital mortality of critical patients treated with IABP. We examined the trend of in-hospital all-cause mortality of critical patients treated with IABP between 1998 and 2008, and surveyed the major diseases of patients treated with IABP.

## Materials and Methods

### Ethics statement

Informed consent was originally obtained by the National Health Research Institutes in Taiwan. To avoid the potential for ethical violations related to the data, the privacy of each individual’s information was protected by de-identifying the data. Thus, informed consent was not required, and an exemption was obtained from the institutional review board of Chi Mei Medical Center (IRB No. 10402-E01).

### Database and patient identification

We used Taiwan’s National Health Insurance Research Database (NHIRD) to do a nationwide study on all patients treated with IABP. The NHIRD consists of detailed healthcare claims data from more than 23 million enrollees, more than 99% of Taiwan’s population [13]. The details of the NHIRD have been described in hundreds of published papers [14]. In our 11-year (1998–2008) retrospective population-based study, inpatient expenditures for hospital admissions were examined to determine the in-hospital mortality of patients treated with IABP. We identified all hospitalized patients treated with IABP from the first two International Classification of Diseases, Ninth Revision, Clinical Modification (ICD-9-CM) diagnosis codes. ICD-9-CM codes were also used to identify patients treated with IABP who underwent PCI or CABG, and to identify patients treated with IABP who had baseline comorbidities of hypertension (HTN) (ICD-9-CM: 401–405), diabetes mellitus (DM) (ICD-9-CM: 250), hyperlipidemia (ICD-9-CM: 272), myocardial infarction (MI) (ICD-9-CM: 410), and cerebrovascular attack (CVA) (ICD-9-CM: 430–438). The comorbidities were selected based on the frequency of their appearance. Age, gender, urbanization level, hospital level, number of days in the intensive care unit (ICU), length of hospitalization, and duration of IABP treatment were also analyzed.

The level of urbanization of Taiwan townships is based on a Taiwanese paper [15] that includes index variables of population density, ratios of the educational levels of people with a college education or above, the ratio of elderly (> 65 years) people, the ratio of people employed as agriculture workers, and the number of physicians per 100,000 people. In-hospital mortality was defined as the number of patients treated with IABP who died before being discharged from the hospital.

### Statistical analysis

The categorical variables that are presented as frequencies and percentages were compared using Pearson's χ^2^ test. Continuous variables, presented as means ± standard deviation (SD) or medians with an interquartile range, were compared using Student’s *t* test or the Wilcoxon rank-sum test. The Cochran-Armitage trend test was used for categorical data analysis to test the association of trends in proportions over specific periods. Cumulative mortality during the first 90 days of each patient’s hospital stay was characterized using Kaplan-Meier plots, with the log-rank test used for the comparisons between genders and between different age groups. SAS 9.3 (SAS Institute, Cary, NC) was used for all statistical analyses but the Kaplan-Meier curves, which were plotted using STATA 12 (Stata Corp., College Station, TX). Significance was set at p < 0.05 (two-tailed).

## Results

We identified 9952 hospitalized patients (mean age: 65.22 ± 12.74 years; almost half [n = 4870: 48.93%] were 65–80 years old) who had been treated with IABP between 1998 and 2008. Most (71.81%) were men (n = 7147). The overall in-hospital mortality during the study period was 13.84% (n = 1377); the highest mortality rates were for patients [a] < 35 years = 39/224 (17.41%) and [b] ≥ 80 years = 172/853 (20.16%). Urbanization level, length of hospital stay, and length of ICU stay were positively associated with mortality. Whether patients were treated in medical centers had no effect on the in-hospital mortality rate. The mean duration of IABP treatment was 3.86 ± 7.39 days. Patients who died in the hospital had been treated with IABP for a longer time (p < 0.0001) (Table 1).

**Table 1:** Demographic characteristics and in-hospital mortality of patients treated with IABP, 1998–2008.

		Died	Survived	
	Overall	(n = 1377)	(n = 8575)	
Characteristics	(n = 9952)	(13.84%)	(86.16%)	p
Age (years) (mean ± SD)	65.22 ± 12.74	66.39 ± 13.99	65.04 ± 12.52	0.0007
Age [n (%)]				
< 35	224 (2.25)	39 (2.83)	185 (2.16)	< 0.0001
35–50	949 (9.54)	146 (10.60)	803 (9.36)	
50–65	3056 (30.71)	323 (23.46)	2733 (31.87)	
65–80	4870 (48.93)	697 (50.62)	4173 (48.66)	
≥ 80	853 (8.57)	172 (12.49)	681 (7.94)	
Gender [n (%)]				
Male	7147 (71.81)	973 (70.66)	6174 (72.00)	0.3052
Female	2805 (28.19)	404 (29.34)	2401 (28.00)	
Hospital level [n (%)]				
Medical Center	7761 (77.98)	1087 (78.94)	6674 (77.83)	0.3567
Regional hospital/Local hospital	2191 (22.02)	290 (21.06)	1901 (22.17)	
Urbanization [n (%)]				
1	4580 (46.02)	671 (48.73)	3909 (45.59)	< 0.0001
2	4799 (48.22)	662 (48.08)	4137 (48.24)	
≥ 3	573 (5.76)	44 (3.20)	529 (6.17)	
ICU days [median (IQR)]	8 (5–17)	8 (3–19)	8 (5–17)	0.0002
Days hospitalized [median (IQR)]	21 (13–33)	13 (5–24)	22 (14–34)	< 0.0001
Duration of IABP (days)	3.86 ± 7.39	4.94 ± 8.05	3.69 ± 7.26	< 0.0001
(mean ± SD)				

Abbreviations: IABP, intra-aortic balloon pumping; ICU, intensive care unit; IQR, interquartile range.

*p from Student’s *t* test or the Wilcoxon test for continuous variables and Pearson's χ^2^ test for categorical variables.

A baseline clinical history of the patients who had been treated with IABP showed HTN (n = 2696; 27.09%), DM (n = 2381; 23.92%), and a prior MI (n = 1538; 15.45%), prior CVA (n = 706; 7.09%), prior heart failure (n = 1904; 19.13%), prior CABG (n = 92; 0.92%) surgery, and prior PCI (n = 22; 0.22%) (Table 2). Moreover, only a history of PCI showed a significant (p = 0.0145) difference between in-hospital mortality and survival.

**Table 2:** The baseline characteristics of underlying diseases in patients treated with IABP, 1998–2008.

		Died	Survived	
	Overall	(n = 1377)	(n = 8575)	
Variable	(n = 9952)	(13.84%)	(86.16%)	p
Hypertension	2696 (27.09%)	359 (26.07%)	2337 (27.25%)	0.3594
Diabetes mellitus	2381 (23.92%)	312 (22.66%)	2069 (24.13%)	0.2352
Prior MI	1538 (15.45%)	190 (13.80%)	1348 (15.72%)	0.0670
Prior CVA	706 (7.09%)	97 (7.04%)	609 (7.10%)	0.9382
Heart failure	1904 (19.13%)	265 (19.24%)	1639 (19.11%)	0.9086
Prior CABG	92 (0.92%)	18 (1.30%)	74 (0.86%)	0.1099
Prior PCI	22 (0.22%)	7 (0.51%)	15 (0.17%)	0.0145

Data are number (percentage) unless otherwise indicated.

Abbreviations: IABP, intra-aortic balloon pumping; MI, myocardial infarction; CVA, cerebrovascular attack, CABG, coronary artery bypass grafting surgery; PCI, percutaneous coronary intervention.

*p from Pearson's χ^2^ test for categorical variables.

**Fig 1:**
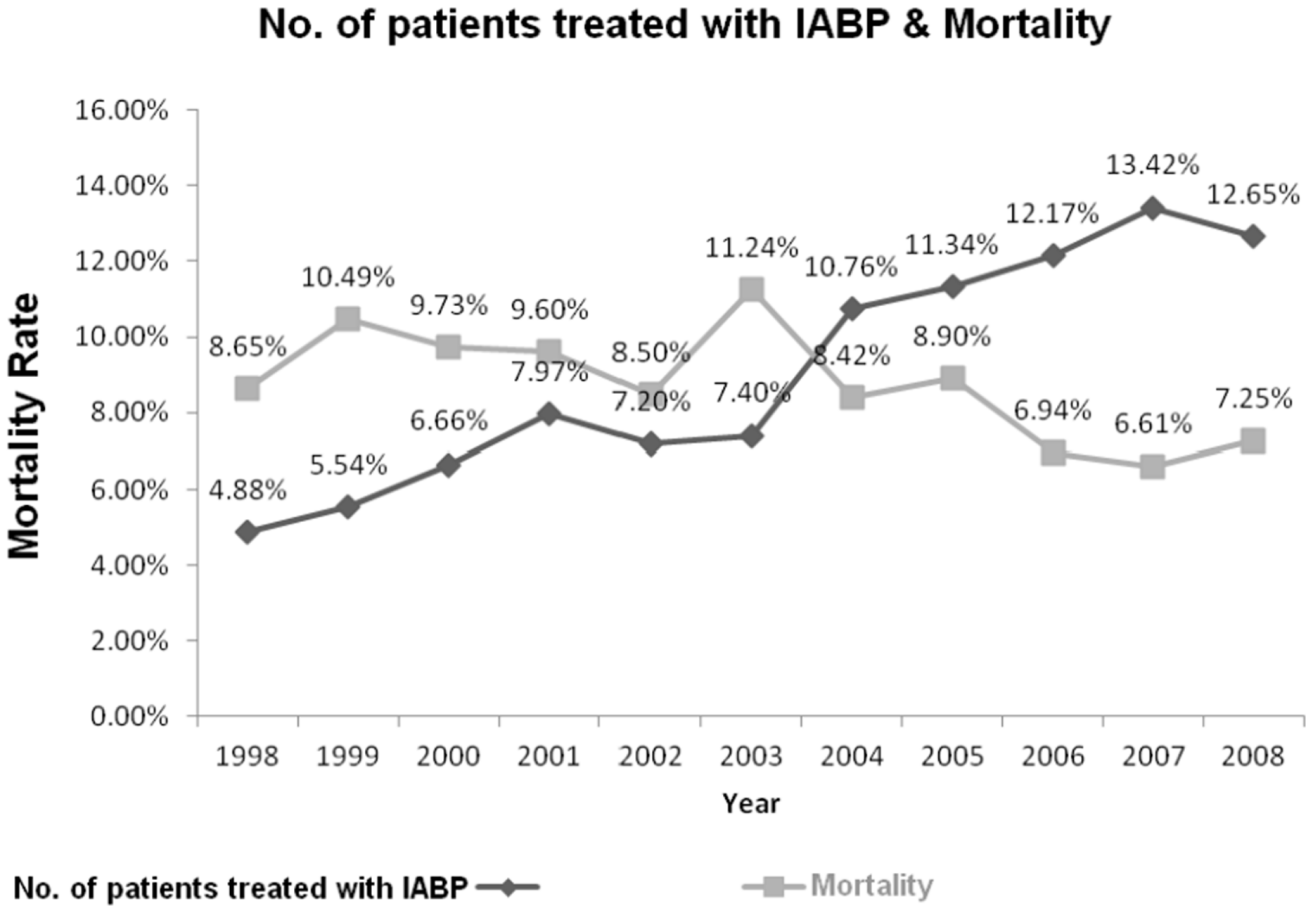
Trends of intra-aortic balloon pumping and in-hospital all-cause mortality, 1998 to 2008. Abbreviation: IABP, intra-aortic balloon pumping. The mortality rate was calculated by dividing [the number of patients treated with IABP who died in a particular year] by [the total number of patients treated with IABP in that particular year]. * p = trends over time: the number of patients treated with intra-aortic balloon pumping (IABP), p < 0.0001; in-hospital all-cause mortality, p = 0.0243.

IABP use in Taiwan has been increasing annually since 1998, but the in-hospital mortality rate per IABP has been decreasing annually since 1999, except for a spike because of the severe acute respiratory syndrome (SARS) in 2003 (Fig 1). The annual in-hospital mortality rate of patients treated with IABP in a particular year fell from 10.49% in 1999 to 7.25% in 2008.

**Fig 2:**
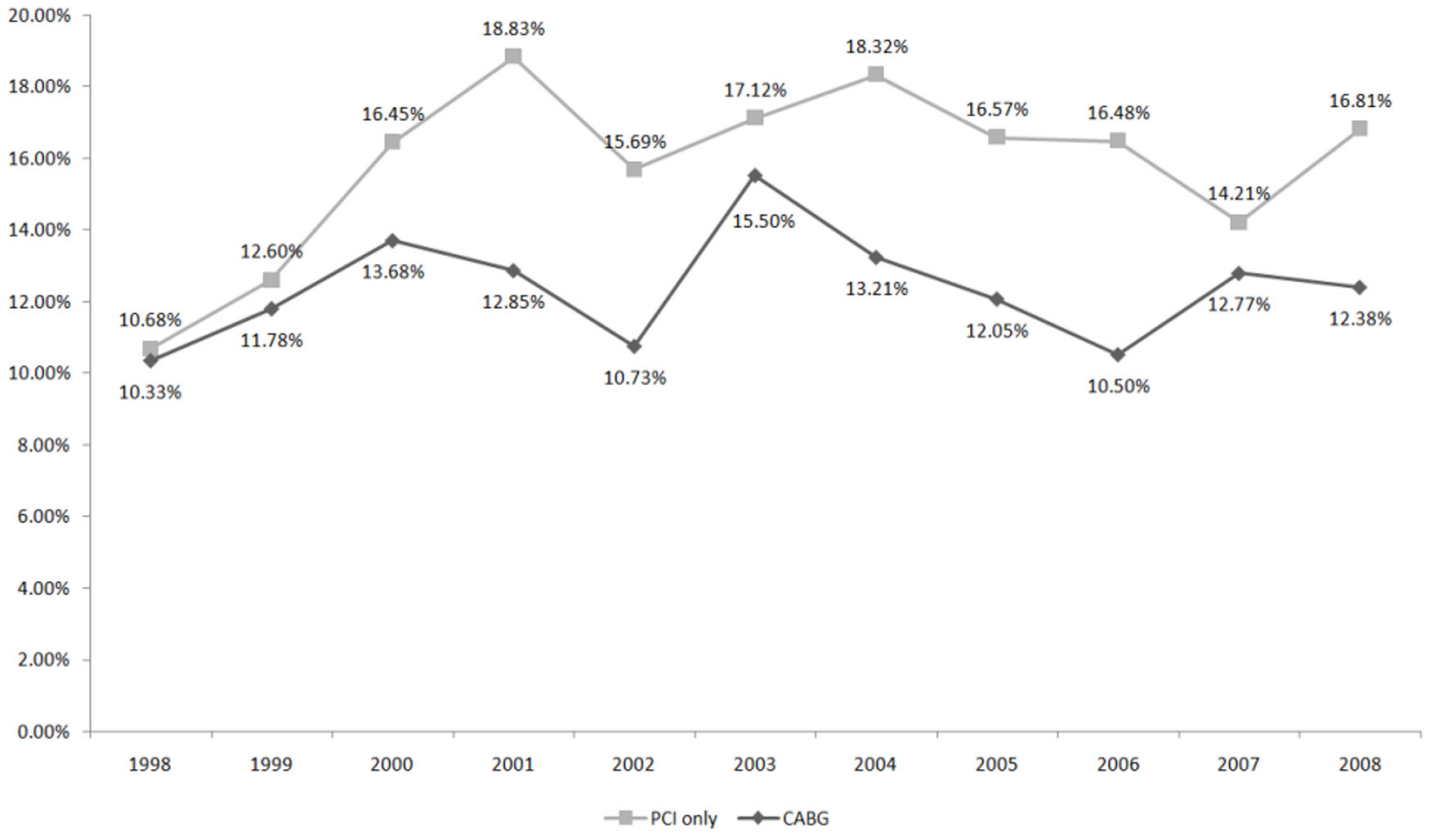
Trends of in-hospital all-cause mortality in patients treated with IABP combined with coronary revascularization, 1998–2008. Abbreviations: IABP, intra-aortic balloon pumping; PCI, percutaneous coronary intervention; CABG, coronary artery bypass grafting surgery. * The mortality rate was calculated by dividing [the number of patients who underwent PCI or CABG, were treated with IABP, and died in a particular year] by [the total number of patients who underwent PCI or CABG and were treated with IABP in that particular year]. * p = trends of in-hospital all-cause mortality over time: PCI group, p = 0.1767; CABG group, p = 0.7501.

**Fig 3:**
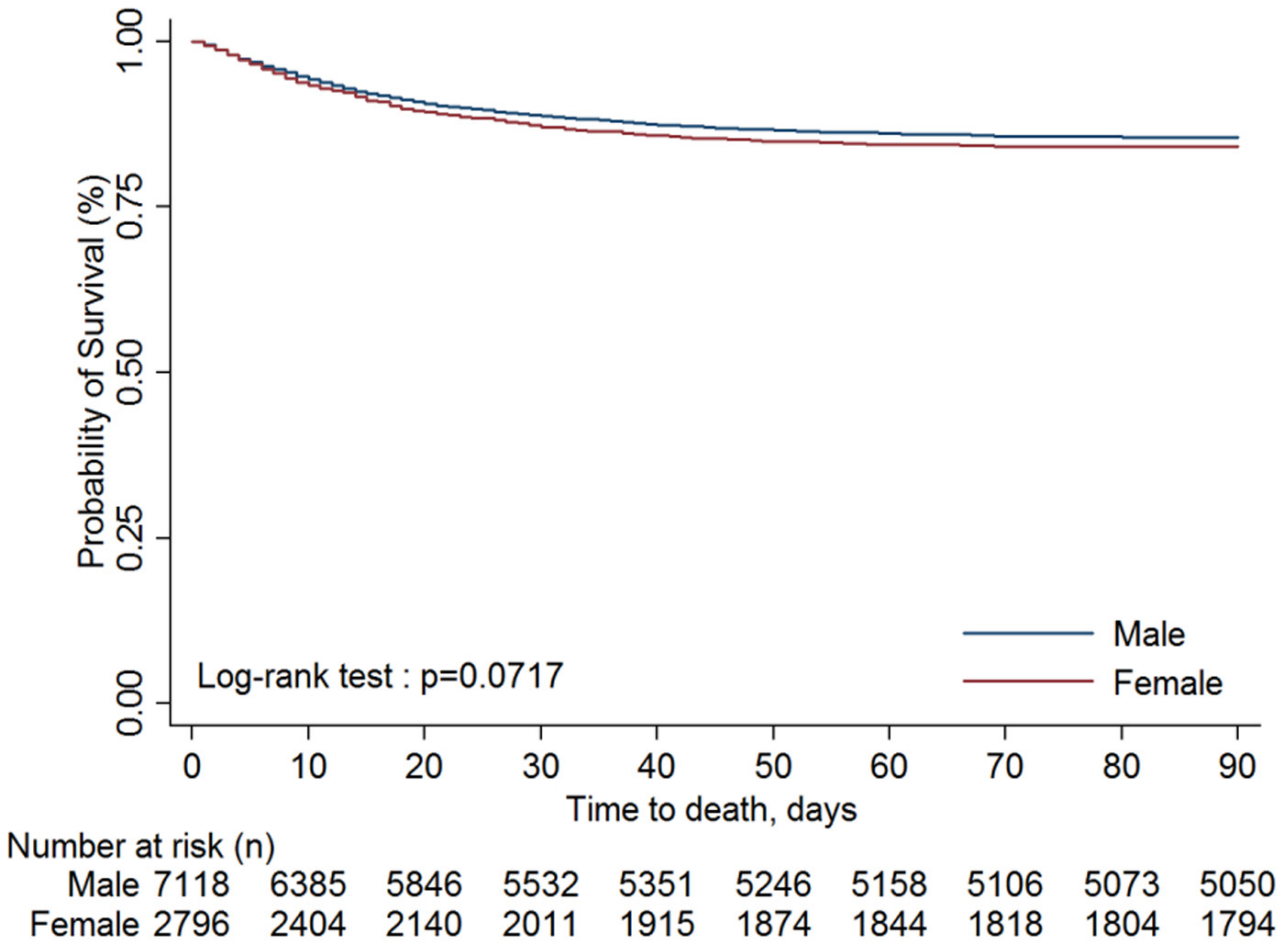
Time-to-event curves by genders for all-cause mortality up to 90 days. The event rate represents Kaplan-Meier estimates; log-rank test: p = 0.0717.

**Fig 4:**
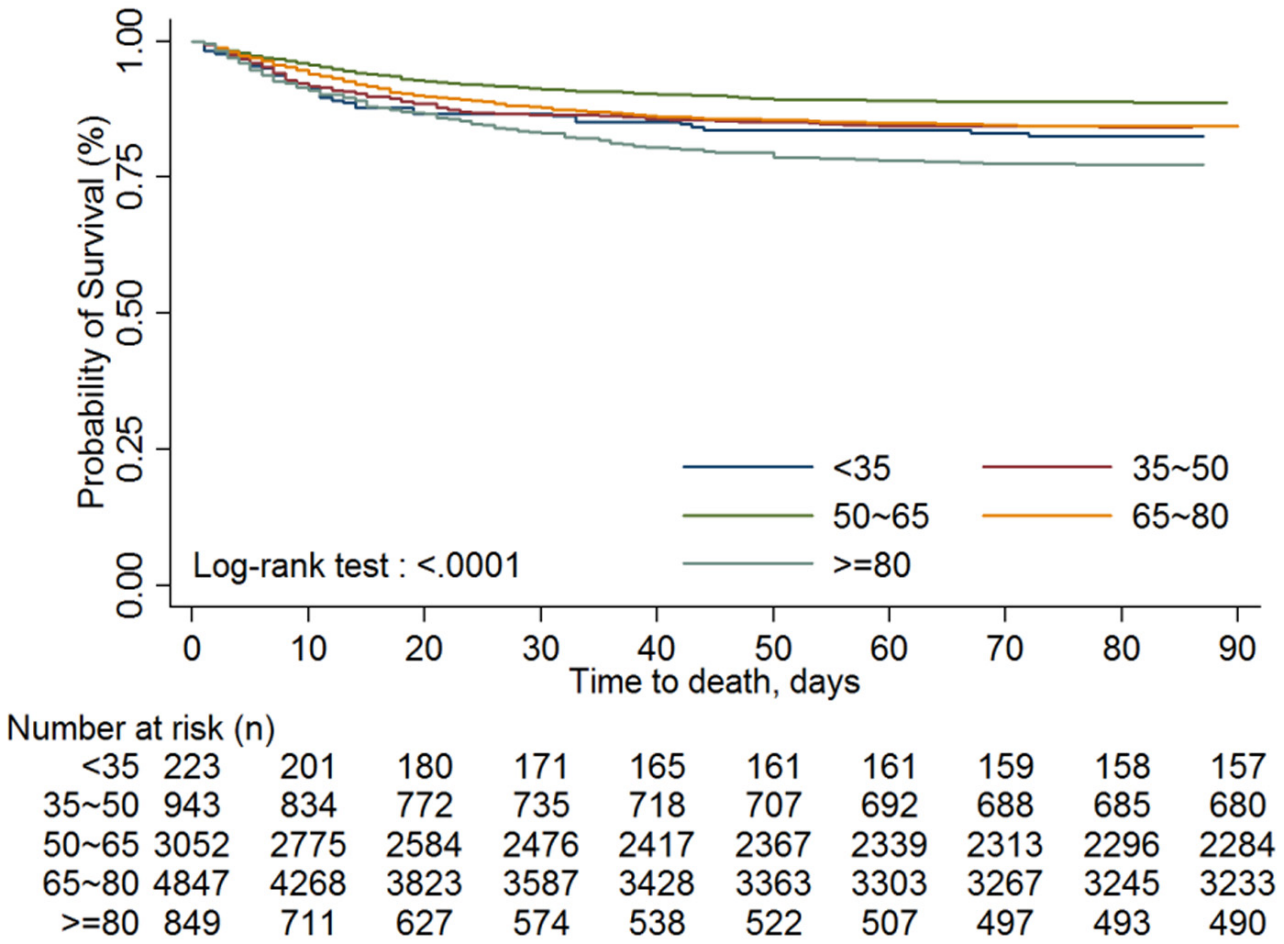
Time-to-event curves by different age groups for all-cause in-hospital mortality up to 90 days. The event rate represents Kaplan-Meier estimates; log-rank test: p < 0.0001.

There were two in-hospital mortality trends (Fig 2), one for patients who underwent CABG+IABP (p_trend_ = 0.7501) in each particular year, and one for patients who underwent PCI+IABP (p_trend_ = 0.1767) in each particular year. In addition, the in-hospital mortality of patients treated with IABP did not differ significantly between genders at 3 months (log-rank test p = 0.0717) (Fig 3). However, patients < 35 years survived for a mean of 3 months longer than did those in other age groups (log-rank test p < 0.0001) (Fig 4).

The in-hospital mortality in patients with AMI and CS was 24.18% (Table 3). All the patients with AMI and CS treated with IABP were retrospectively divided into three groups: IABP-only, PCI+IABP, and CABG+IABP. In-hospital mortality rates were not significantly different between the three groups: IABP-only was 7/35 (20.00%); CABG+IABP was 114/453 (25.17%); and PCI+IABP was 12/62 (19.35%). The in-hospital mortality of patients who had been treated with CABG and IABP in the same admission was 12.27%. Patients who had been treated with CABG and IABP for 1 vessel in the same admission had a higher in-hospital mortality rate of 16.53%. The mortality in patients who had been treated with IABP and PCI for 1 vessel, 2 vessels, or 3 vessels in the same admission was not significantly different. The in-hospital mortality of patients who had been treated with PCI-only plus IABP was 15.45%.

**Table 3:** In-hospital all-cause mortality rates in patients treated with IABP who had AMI and CS, and patients treated with IABP who underwent coronary revascularization, 1998–2008.

No. of patients	Overall	In-hospital mortality rate	p
AMI with CS	550	133 (24.18%)	
Treated with:			
Medication	35	7 (20.00%)	0.5064
PCI	62	12 (19.35%)	
CABG	453	114 (25.17%)	
CABG			
Total	7635	937 (12.27%)	
for one-vessel	514	85 (16.53%)	< 0.0001
for two vessels	1453	221 (15.20%)	
for three vessels	5668	631 (11.13%)	
PCI			
Total	576	89 (15.45%)	
for one vessel	413	69 (16.71%)	0.4238
for two vessels	130	17 (13.08%)	
for three vessels	33	3 (9.09%)	

Data are n (%) unless otherwise indicated. Mortality rate: [No. of deaths]/[No. of patients].

Abbreviations: IABP, intra-aortic balloon pumping; AMI, acute myocardial infarction; CS, cardiogenic shock; CABG, coronary artery bypass grafting surgery; PCI, percutaneous coronary intervention.

*p from Pearson's χ^2^ test for categorical variables.

Based on their first two ICD-9-CM discharge diagnosis codes, six major comorbid diseases for hospitalized patients treated with IABP were identified: acute coronary syndrome (ACS), CS, heart failure, fetal cardiac arrhythmia, acute myocarditis, and valvular heart disease (Table 4). Patients with acute myocarditis who were being treated with IABP had the highest mortality rate: 26.47%. The in-hospital mortality of patients with ACS was 15.14%, and of patients with CS was 22.14%.

**Table 4:** In-hospital mortality rates of patients treated with IABP. Stratified based on the first two ICD-9-CM discharge diagnosis codes.

Major diagnosis	No. of patients	In-hospital mortality rate [n (%)]
Acute coronary syndrome	4043	612 (15.14%)
Cardiogenic shock	944	209 (22.14%)
Heart failure	1991	148 (14.93%)
Fetal cardiac arrhythmia	97	21 (21.65%)
Acute myocarditis	68	18 (26.47%)
Valvular heart disease	1304	189 (14.49%)

Abbreviations: IABP, intra-aortic balloon pumping; ICD-9-CM, International Classification of Diseases, Ninth revision, Clinical Modification.

## Discussion

Our large-population cohort study found that the in-hospital mortality per given IABP treatment had fallen annually between 1998 and 2008 in Taiwan despite an annual increase in the number of patients treated with IABP. However, the in-hospital mortality was higher for patients treated with coronary revascularization plus IABP, which was contrary to the overall trend in patients treated with IABP during this period.

More men than women in our study had been treated with IABP, which is consistent with other studies [7,11]. The in-hospital all-cause mortality for patients treated with IABP between 1998 and 2008 was 13.8%, significantly lower than the 20.1% in America and 28.7% in Europe between 1997 and 2002 [16]. Lower in-hospital mortality in our study might be attributable to improvements in the medical care given to patients in Taiwan, to the medications used, or to different conditions for using IABP during these years. We also found that shorter hospitalizations and longer durations of IABP use were associated with higher mortality. There were no hospital-level-based differences in the in-hospital mortality rates of patients treated with IABP in our study, which is consistent with a National Cardiovascular Data Registry study [17]. In the Benchmark Registry study [11], women had a greater risk for major complications from IABP. Patients > 80 years old treated with IABP had the highest in-hospital mortality rate. The in-hospital mortality in patients < 35 years old treated with IABP was the second highest, but their 3-month mortality rate was the lowest of all age groups. This is probably because patients < 35 years old had fewer comorbid diseases.

There is no consensus on the timing of removing intra-aortic balloon pumps. Bignami et al. [18] indicated that most intra-aortic balloon pumps can be removed in the first two days (77%). In our study, the average duration of IABP treatment was 3.86 ± 7.39 days. The duration for patients who died in the hospital was significantly longer than for patients who survived (4.94 ± 8.05 vs. 3.69 ± 7.26 days, respectively; p < 0.0001). Our findings confirmed the claim [18] that the timing of removing intra-aortic balloon pumps depends upon the medical condition of the patient or the protocols of the hospital. In addition, we offer new information in a different area of IABP management on the timing of IABP removal.

Chen et al. [19] reported a higher risk of mortality in people with ischemic heart disease in less urbanized areas in Taiwan, and another study [20] showed higher mortality for coronary heart disease in rural areas in the United States. We also investigated the association between urbanization and the in-hospital mortality of patients treated with IABP. We found that, in the less urbanized areas in Taiwan, patients treated with IABP had higher in-hospital mortality, probably because there are fewer healthcare resources there and because access to those healthcare resources is not as rapid or easy as it is in more urbanized areas.

In the present study, ICD-9-CM discharge diagnosis codes showed that IABP was most frequently used in patients with CAD. Most patients (n = 7635/9952, 76.7%) had been treated with IABP and CABG during the same admission; therefore, it was assumed that most patients required IABP to be weaned from a CPB, not to treat CS. Benchmark Registry data from 1996 to 2000 [11] showed that IABP was most often used to provide hemodynamic support during or after cardiac catheterization (20.6%). Our study showed that the in-hospital all-cause mortality rate of patients treated with CABG+IABP during the same hospitalization was 12.27% and that there was a similar mortality rate (12.6%) between 1996 and 2001 reported by Hemo et al. [21] and a higher in-hospital mortality rate (16.8%) in the Benchmark Registry of 1996–2000 [11]. In addition, this suggests a higher risk of in-hospital mortality when PCI is combined with IABP: our rate for these patients was 15.45%. Another recent large nationwide study [12] reported that the in-hospital mortality of patients in the USA treated with PCI plus IABP from 1998 to 2008 was 20.31%, with a decline to 17.8% in 2008. The early Benchmark Registry data [11] reported an in-hospital mortality rate of 18.4% for patients who underwent PCI combined with IABP. A recent balloon pump-assisted coronary intervention study [22] reported a 15.2% rate for major adverse cardiac and cardiovascular events in patients undergoing high-risk PCI combined with IABP.

The in-hospital mortality rate for our 944 patients with CS who were treated with IABP was 22.1%, the incidence rate of AMI comorbid with CS in patients with ACS was 13.60% (550/4043), and the 24.18% in-hospital mortality rate in patients with AMI comorbid with CS was much lower than in other studies [23–25]. Babaev et al. [25] reported that the 2004 in-hospital mortality rate of patients with AMI comorbid with CS, and listed in the National Registry of Myocardial Infarction (NRMI), was 47.9%; for patients who underwent primary PCI, it was 33.9%; and for patients who underwent CABG, it was 16.7%. From the Benchmark Registry database [23], the in-hospital mortality in patients with CS treated with IABP was 42.0%. We think there were two possible reasons for the lower mortality rate of patients with AMI and comorbid CS in our study. Firstly, emergency revascularization using PCI or CABG is done in Taiwan for most patients with STEMI and comorbid CS based on the current American College of Cardiology Foundation/American Heart Association (ACCF/AHA) guidelines [10] and on our customary daily practice. Of 550 patients with AMI and CS treated with IABP in our study, 453 (82.36%) had undergone CABG, 62 (11.27%) had undergone PCI, and only 35 (6.36%) had been treated only with medication. Therefore, most patients with AMI and comorbid CS treated with IABP underwent CABG, and our in-hospital mortality rate was 25.16%. Hemo et al. [21] reported that the in-hospital mortality rate of patients with CS undergoing CABG s/p IABP was 22.2%. Secondly, AMI comorbid with CS in our study was considered a major disease. We excluded 1390 patients because AMI with CS was not their major diagnosis, and because these patients had a higher in-hospital mortality rate and they had other comorbidities.

This study has some limitations. First, this is a retrospective analysis based on data from a health insurance claims database. All the diagnoses were based on the ICD-9-CM codes, not patients’ medical charts or personal observation by the authors. The NHIRD provides no detailed hemodynamic data, pathological data, or ratings of disease severity. The IABP-related complications, such as vascular and septic complications [26], and the cause of death are not inferable from claims records. In addition, in the study we focus on AMI with CS as a major diagnosis based on the first two ICD-9-CM code discharge diagnoses. Some patients with AMI were not included in the study because CS was not one of their first two ICD-9-CM codes. Thus, we cannot exclude the possibility that unmeasured factors may have confounded the results. Second, because we could not obtain the patients’ medical charts, we did not know precisely why each patient was treated with IABP. However, because we used the first two ICD-9-CM codes to detect each patient’s main diseases, we are confident that they were the most likely reasons for IABP treatment. Third, we were unable to determine whether the PCI, CABG, and IABP treatments were before, during, or after cardiac catheterization.

## Conclusions

The in-hospital mortality rate of IABP treatment decreased annually in Taiwan, but the trend of in-hospital mortality in those patients who underwent coronary revascularization did not present a similar trend. These high-risk patients who underwent coronary revascularization with IABP had a higher and unstable in-hospital mortality rate. This study offers valuable information about mortality after using IABP in Taiwan.

## References

[1] Zangrillo A, Pappalardo F, Dossi R, Di Prima AL, Sassone ME, Greco T, et al Preoperative intra-aortic balloon pump to reduce mortality in coronary artery bypass graft: a meta-analysis of randomized controlled trials. Crit Care. 2015;19: 10 10.1186/s13054-014-0728-1 25588568 PMC4316767

[2] Kapelios CJ, Terrovitis JV, Siskas P, Kontogiannis C, Repasos E, Nanas JN Counterpulsation: a concept with a remarkable past, an established present and a challenging future. Int J Cardiol. 2014;172: 318–325. 10.1016/j.ijcard.2014.01.098 24525157

[3] Barron HV, Every NR, Parsons LS, Angeja B, Goldberg RJ, Gore JM, et al The use of intra-aortic balloon counterpulsation in patients with cardiogenic shock complicating acute myocardial infarction: data from the National Registry of Myocardial Infarction 2. Am Heart J. 2001;141: 933–939. 11376306 10.1067/mhj.2001.115295

[4] Sanborn TA, Sleeper LA, Bates ER, Jacobs AK, Boland J, et al Impact of thrombolysis, intra-aortic balloon pump counterpulsation, and their combination in cardiogenic shock complicating acute myocardial infarction: a report from the SHOCK Trial Registry. J Am Coll Cardiol. 2000;36: 1123–1129. 10985715 10.1016/s0735-1097(00)00875-5

[5] Theologou T, Bashir M, Rengarajan A, Khan O, Spyt T, Richens D, et al Preoperative intra aortic balloon pumps in patients undergoing coronary artery bypass grafting. Cochrane Database Syst Rev. 2011;(1): CD004472 10.1002/14651858.CD004472.pub3 21249662 PMC8094869

[6] Landoni G, Rodseth RN, Santini F, Ponschab M, Ruggeri L, Székely A, et al Randomized evidence for reduction of perioperative mortality. J Cardiothorac Vasc Anesth. 2012;26: 764–772. 10.1053/j.jvca.2012.04.018 22726656

[7] Thiele H, Zeymer U, Neumann FJ, Ferenc M, Olbrich HG, Hausleiter J, et al Intraaortic balloon support for myocardial infarction with cardiogenic shock. N Engl J Med. 2012;367: 1287–1296. 10.1056/NEJMoa1208410 22920912

[8] Thiele H, Zeymer U, Neumann FJ, Ferenc M, Olbrich HG, Hausleiter J, et al Intra-aortic balloon counterpulsation in acute myocardial infarction complicated by cardiogenic shock (IABP-SHOCK II): final 12 month results of a randomised, open-label trial. Lancet. 2013;382: 1638–1645. 10.1016/S0140-6736(13)61783-3 24011548

[9] Task Force on the management of ST-segment elevation acute myocardial infarction of the European Society of Cardiology (ESC), Steg PG, James SK, Atar D, Badano LP, Blömstrom-Lundqvist C, et al ESC Guidelines for the management of acute myocardial infarction in patients presenting with ST-segment elevation. Eur Heart J. 2012;33: 2569–2619. 10.1093/eurheartj/ehs215 22922416

[10] American College of Emergency Physicians; Society for Cardiovascular Angiography and Interventions, O'Gara PT, Kushner FG, Ascheim DD, Casey DE Jr, Chung MK, et al 2013 ACCF/AHA guideline for the management of ST-elevation myocardial infarction: executive summary: a report of the American College of Cardiology Foundation/American Heart Association Task Force on Practice Guidelines. J Am Coll Cardiol. 2013;61: e78–e140. 10.1016/j.jacc.2012.11.019 23256914

[11] Ferguson JJ 3rd, Cohen M, Freedman RJ Jr, Stone GW, Miller MF, Joseph DL, et al The current practice of intra-aortic balloon counterpulsation: results from the Benchmark Registry. J Am Coll Cardiol. 2001;38: 1456–1462. 11691523 10.1016/s0735-1097(01)01553-4

[12] Patel H, Shivaraju A, Fonarow GC, Xie H, Gao W, Shroff AR, et al Temporal trends in the use of intraaortic balloon pump associated with percutaneous coronary intervention in the United States, 1998–2008. Am Heart J. 2014;168: 363–373.e12 10.1016/j.ahj.2014.02.015 25173549 PMC4155763

[13] Cheng CL, Kao YH, Lin SJ, Lee CH, Lai ML. Validation of the National Health Insurance Research Database with ischemic stroke cases in Taiwan. Pharmacoepidemiol Drug Saf. 2011;20: 236–242. 10.1002/pds.2087 21351304

[14] Chen YC, Yeh HY, Wu JC, Haschler I, Chen TJ, Wetter T. Taiwan’s National Health Insurance Research Database: administrative health care database as study object in bibliometrics. Scientometrics. 2011;86: 365–380. 10.1007/s11192-010-0289-2

[15] Liu CY HY, Chuang YL, Chen YJ, Weng WS, Liu JS, Liang KY. Incorporating development stratification of Taiwan townships into sampling design of large scale health interview survey. J Health Manag. 2006;4: 1–22.

[16] Cohen M, Urban P, Christenson JT, Joseph DL, Freedman RJ Jr, Miller MF, et al Intra-aortic balloon counterpulsation in US and non-US centres: results of the Benchmark Registry. Eur Heart J. 2003;24: 1763–1770. 14522572 10.1016/j.ehj.2003.07.002

[17] Curtis JP, Rathore SS, Wang Y, Chen J, Nallamothu BK, Krumholz HM. Use and effectiveness of intra-aortic balloon pumps among patients undergoing high risk percutaneous coronary intervention: insights from the National Cardiovascular Data Registry. Circ Cardiovasc Qual Outcomes. 2012;5: 21–30. 10.1161/CIRCOUTCOMES.110.960385 22147887 PMC3801197

[18] Bignami E, Tritapepe L, Pasin L, Meroni R, Corno L, Testa V, et al A survey on the use of intra-aortic balloon pump in cardiac surgery. Ann Card Anaesth. 2012;15: 274–277. 10.4103/0971-9784.101871 23041684

[19] Chen BK, Yang CY. Differences in age-standardized mortality rates for avoidable deaths based on urbanization levels in Taiwan, 1971–2008. Int J Environ Res Public Health. 2014;11: 1776–1793. 10.3390/ijerph110201776 24503974 PMC3945567

[20] Kulshreshtha A, Goyal A, Dabhadkar K, Veledar E, Vaccarino V. Urban-rural differences in coronary heart disease mortality in the United States: 1999–2009. Public Health Rep. 2014;129: 19–29. 10.1177/003335491412900105 PMC3863000 24381356

[21] Hemo E, Medalion B, Mohr R, Paz Y, Kramer A, Uretzky G, et al Long-term outcomes of coronary artery bypass grafting patients supported preoperatively with an intra-aortic balloon pump. J Thorac Cardiovasc Surg. 2014;148: 1869–1875. 10.1016/j.jtcvs.2013.12.063 24521970

[22] Perera D, Stables R, Thomas M, Booth J, Pitt M, Blackman D, et al Elective intra-aortic balloon counterpulsation during high-risk percutaneous coronary intervention: a randomized controlled trial. JAMA. 2010;304: 867–874. 10.1001/jama.2010.1190 20736470

[23] Urban PM, Freedman RJ, Ohman EM, Stone GW, Christenson JT, Cohen M, et al In-hospital mortality associated with the use of intra-aortic balloon counterpulsation. Am J Cardiol. 2004;94: 181–185. 15246896 10.1016/j.amjcard.2004.03.058

[24] Goldberg RJ, Spencer FA, Gore JM, Lessard D, Yarzebski J. Thirty-year trends (1975 to 2005) in the magnitude of, management of, and hospital death rates associated with cardiogenic shock in patients with acute myocardial infarction: a population-based perspective. Circulation. 2009;119: 1211–1219. 10.1161/CIRCULATIONAHA.108.814947 19237658 PMC2730832

[25] Babaev A, Frederick PD, Pasta DJ, Every N, Sichrovsky T, Hochman JS, et al Trends in management and outcomes of patients with acute myocardial infarction complicated by cardiogenic shock. JAMA. 2005;294: 448–454. 16046651 10.1001/jama.294.4.448

[26] Severi L, Vaccaro P, Covotta M, Landoni G, Lembo R, Menichetti A. Severe intra-aortic balloon pump complications: a single-center 12-year experience. J Cardiothorac Vasc Anesth. 2012;26: 604–607. 10.1053/j.jvca.2012.01.037 22445181

